# Centering Indigenous Voices: The Role of Fire in the Boreal Forest of North America

**DOI:** 10.1007/s40725-022-00168-9

**Published:** 2022-07-27

**Authors:** Amy Cardinal Christianson, Colin Robert Sutherland, Faisal Moola, Noémie Gonzalez Bautista, David Young, Heather MacDonald

**Affiliations:** 1 Canadian Forest Service, Natural Resources Canada, Northern Forestry Centre, 5320 – 122 St, Edmonton, Edmonton, AB T6H 3S5 Canada; 2 National Fire Management Division, Natural Resource Management Branch, Parks Canada, Rocky Mountain House National Historic Site, Rocky Mountain House, T4T 2A4 Canada; 3 Conservation Through Reconciliation Partnership, Department of Geography, Environment & Geomatics, University of Guelph, 350 Hutt Building, Guelph, ON N1G2W1 Canada; 4 Department of Geography, Environment and Geomatics, University of Guelph, 350 Hutt Building, Guelph, ON N1G2W1 Canada; 5 Centre Interuniversitaire d’études et de recherches autochtones (CIÉRA), Université Laval, Québec, Canada; 6 Canadian Forest Service, Great Lakes Forestry Centre, Natural Resources Canada, 1219 Queen Street, Sault Ste. Marie, Sault Ste. Marie, ON P6A 2E5 Canada

**Keywords:** Indigenous fire knowledge, Cultural burning, Cultural fire, Prescribed burning, Colonialism

## Abstract

**Purpose of Review:**

Indigenous perspectives have often been overlooked in fire management in North America. With a focus on the boreal region of North America, this paper provides a review of the existing literature documenting Indigenous voices and the historical relationship of Indigenous peoples in northern North America to fire and landscapes that burn.

**Recent Findings:**

Early research on the topic explored how Indigenous people used fire in the boreal forest, with most research coming out of case studies in northern Alberta. Emerging research in the last two decades has broadened the geographic focus to include case studies in Alaska, Ontario, Labrador, and other regions in North America. This broadening of focus has shown that the diversity of Indigenous peoples in North America is reflected in a diversity of relationships to fire and landscapes that burn. Of note is an emerging interest in Indigenous fire knowledge in the wake of settler colonialism.

**Summary:**

Indigenous peoples in the boreal forest have applied fire on their landscapes to fulfill numerous objectives for thousands of years. More than a tool, Indigenous peoples in the boreal view fire as an agent, capable of movement, destruction and creation, acting on the landscape to create order, within a living, connected environment. Unfortunately, restrictions on the application of Indigenous fire knowledge and practice initiated during early colonial times remains a contemporary challenge as well.

**Supplementary Information:**

The online version contains supplementary material available at 10.1007/s40725-022-00168-9.

## Introduction

### Background and Purpose of Review

Indigenous Nations in the boreal forest have lived with fire since time immemorial [1•].^1^ As such, Indigenous fire knowledge in what is now Canada and Alaska is not singular, but is plural and deeply connected to place [2]. These Indigenous Nations hold significant knowledge about fire-driven landscapes and enact a set of practices and relationships with landscapes that burn [3•]. Across North America and beyond, Indigenous and non-Indigenous scholars have discussed the value of Indigenous knowledges to wildland fire management and missed opportunity to learn from said communities [1•, 4–8], as well as the important role Indigenous peoples play in stewarding biodiversity [9–17] and nature-based solutions to climate change [18, 19]. While these knowledges may compliment dominant science’s understanding of environments and fire, Indigenous knowledge should be valued in its own right as these knowledge systems are closely tethered to both land and people, providing us with more than a set of universal truths, but ways of living with fire [2, 4–8]. However, much of the literature focus in the boreal biome has been on biophysical research by non-Indigenous researchers who sought to document the roles of fire in the boreal forest without including Indigenous peoples in the research process [20–24]. With this paper, we aim to start changing this trend.

As of 2021, there are over 600 primarily Indigenous communities in the North American boreal biome. These communities are composed of diverse Indigenous Nations, but First Nations represent the largest set of communities living in the region.^2^ These communities are disproportionately impacted by wildfire evacuations and smoke [25, 26] as many are located in rural, sometimes remote, forested regions experiencing the impacts of climate change and policies of attempted fire exclusion resulting in increased fire suppression challenges [27, 28••]. Additionally, wildfire activity in the boreal forest of North America is set to increase from fuel loading [29] and climate change [30]. An appreciation for Indigenous fire knowledge in the boreal forest is growing, which has resulted in a few partnerships between Indigenous Nations and wildland fire management agencies [1•]. However, there is much work still to be done on shifting the power dynamics around wildfire management decision making.

In comparison, in places like Australia, Indigenous fire knowledge is reformulating how the work of fire management is practiced and is creating opportunities for Indigenous and science communities alike [4, 8, 31–34]. These efforts have made specific reference to the role of colonialism in the disruption of Australian fire regimes. Like Australia, colonialism has also impacted fire regimes in the boreal forest in North America. Indigenous fire practices were criminalized by settler governments, and a variety of colonial mechanisms interrupted Indigenous land use practices throughout much of North America [35–37]. The impact of various assimilation efforts on Indigenous Nations, including residential schools, has interrupted or erased the transmission of Indigenous knowledge in general [38, 39].

Only a limited set of scholars have worked with Indigenous peoples to document both the contemporary and historical relationships between Indigenous peoples of the boreal and fire as powerful agents of change across the boreal landscape. To date, there has not been a review of existing literature on fire in the boreal of North America centering Indigenous peoples’ voices. This paper advances the current state of knowledge by assembling a sparse, but vastly diverse literature reflecting Indigenous fire knowledge in the boreal forest. We present more than three dozen species, including vascular plants, insects, and mammals, referenced in the literature as managed by Indigenous peoples using fire. By summarizing common themes in literature that centers Indigenous voices [40], we invite readers to consider how Indigenous fire knowledge could be celebrated and referenced within the wider field of forestry literature. We also argue in this paper that Indigenous fire knowledge is part of a holistic system of ecosystem stewardship and that Indigenous peoples and their knowledge systems should inform changes in wildfire, forest, and protected area management policies and practices in Canada, and the boreal in particular.

### A Conceptual Framework

Humans are notably absent from contemporary fire literature about the boreal region, as Oberndorfer [41•] notes. However, most landscapes in North America have been shaped by Indigenous practices long before European colonization [42, 43] and there are a multiplicity of ways of valuing and relating to so-called “nature.” Those interested in “historical” human relationships to fire must consider what stopped them from being “contemporary” relationships. Historically, by viewing the boreal forest as “empty” rather than tended landscapes, settlers made the argument that land inhabited by Indigenous peoples was free to occupy, using the justifications of the “Doctrine of Discovery” and associated “terra nullius” (empty land) to justify their actions of dispossession and occupation [44, 45]. In reality, Indigenous peoples in the boreal forest have intentionally modified their landscapes for thousands of years, in part through burning practices [9, 11, 36, 37, 41•, 46].

**Fig. 1:**
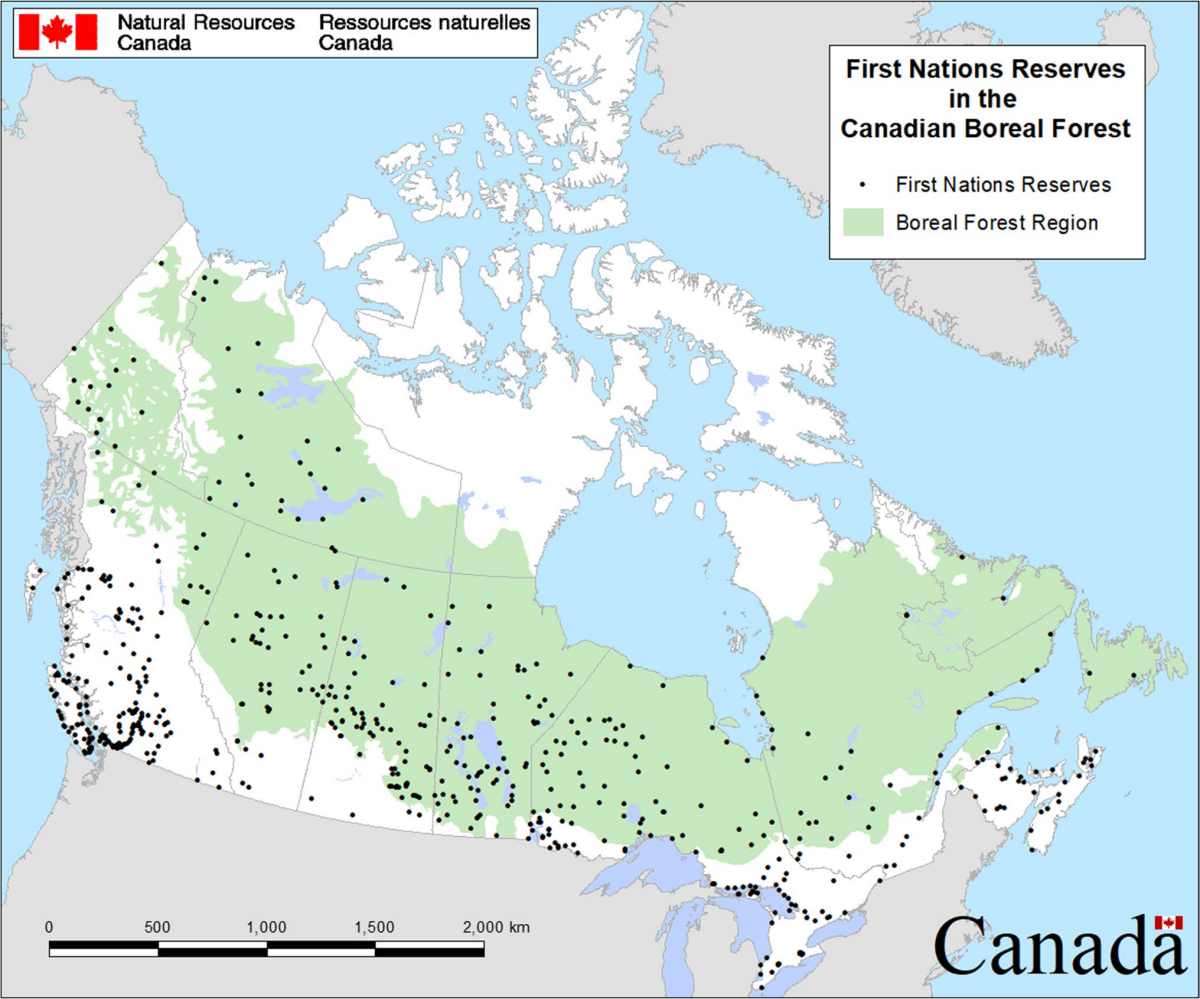
Extent of the boreal forest region and location of First Nations reserves in Canada (courtesy of John M. Little, Natural Resources Canada, Canadian Forest Service, Northern Forestry Centre, Edmonton, Alberta)

Indigenous peoples insist that the boreal has always been tied to people, and should be considered a complex *cultural landscape* (Fig. 1). Following ongoing engagement with Elders and community members in Pikangikum First Nation in what is now northwest Ontario, non-Indigenous scholars have described the boreal forest as an Aboriginal cultural landscape [47, 48]. The definition put forward by Miller and Davidson-Hunt [47] challenged earlier conceptualizations of cultural landscapes, which envisioned landscapes as the result of exclusively human action on the world. Instead, Miller and Davidson-Hunt’s [47] discussions with their Anishinaabe interlocutors challenge this definition by suggesting that humans were not the only agents changing the landscape, but that fire and other beings [49] had the ability to express agency as well:

*Our goal is a more holistic understanding of cultural landscapes informed by our Anishinaabe colleagues and other scholars who suggest that cultural landscapes are both material and symbolic and include a society’s unique worldview, ontology, history, institutions, practices and the networks of relationships between human and nonhuman beings* [47: p. 402]

This definition positions landscapes as the product of both human and nonhuman agents and is reflected in ongoing discussions in anthropology, geography, ethnoecology, and forestry (among others) who conceptualize landscapes not just as ecosystems or ecosystem services, but complex socio-natural assemblages [50, 51]. In that sense, the boreal forest is neither a wild virgin landscape, nor the product of exclusive human enterprise. Further, there is also a rich, geographically diverse literature exploring how fire is a particularly important feature of this place-making relationship [52, 53]. Historical conceptions of the boreal forest as a *wilderness* empty of complex human societies misses both the role that Indigenous peoples played in the boreal landscape, and in particular, the human hand which influenced the expression of fire on the landscape, and human relationships with other nonhuman beings [41•, 54, 55•].

The dominant narrative of wildland fire history in the boreal forest focuses on large-scale periodic stand-replacing fires. Indeed, most published histories of wildland fire gloss over or do not mention frequent small-scale fires, including Indigenous burning, that are often missed by large-scale measurements [21–23, 56–60). Instead, this review invites the reader to consider how other ways of knowing fire and landscapes that burn are worth paying attention to. To understand the boreal forest as a landscape-in-the-making contributes to a wider appreciation for Indigenous knowledge and forms of land care native to this expansive and diverse landscape. This approach also invites a critical analysis of how relationships between Indigenous peoples, fire, and traditional knowledge have been suppressed, interrupted, and erased. This review offers an opportunity to see this region as more than an ecological landscape — but rather an *Indigenous cultural landscape* — by forefronting literature that includes the knowledge and perspectives of Indigenous peoples in the boreal forest of North America.

## Methods

Our author team is made up of Indigenous peoples and allies. We reviewed literature that made explicit reference to: the boreal and Indigenous use of fire and burned sites that included Indigenous voices. This literature is relatively limited, despite a number of calls for more research in the region [28••, 41•, 55•, 61, 62••] and a need to make space for Indigenous scientists in academic publishing [63–65]. The literature is even more limited for the boreal forests of Eastern Canada (Quebec and the Maritime Provinces), and that which exists is often laced in common racist tropes against Indigenous peoples.^3^ An unavoidable limitation of this review is the relatively recent appreciation for the value of Indigenous knowledge within the wider forestry literature. This, coupled with the systemic barriers to Indigenous access to the academy, has translated to very few articles published by Indigenous peoples on this topic, and even fewer by Indigenous peoples documenting the knowledge of their own Nations [55•, 69].^4^

Our review included papers relying on archival and ethnographic research methods, including interviews with Elders, to document fire practices from the perspective of Indigenous peoples. Because this paper concerns Indigenous perspectives about fire, we intentionally did not review publications that hypothesize Indigenous fire use practices based on archeological, dendrochronology, or other methods of assessing fire history that do not engage with Indigenous peoples directly. In terms of limitations, our review does not delve into the impact of fire on wildlife populations; rather, we focus on a subset of culturally significant plants and animals identified by Indigenous peoples that are known to have had their production enhanced with cultural burning. We also do not review all ethnographic accounts of Indigenous engagement with the boreal forest more broadly. Further, this review does not interrogate fire’s role in Indigenous cosmologies or the role of fire in the home.

Much of the research reviewed in this article takes inspiration from the work of anthropologist Henry Lewis and the Dene and Woodland Cree communities he worked with in the 1970s. Lewis was one of the first to document human-fire relationships in the region and in Canada and his research is often referenced by human-fire scholars across the world. We also acknowledge that the “boreal forest” is a concept that deserves further interrogation as it fails to appreciate the many different human and more-than-human relationships at work in this expansive region.

The literature review that follows contrasts the focus of early anthropological work, which portrayed Indigenous use of fire as a tool, with later studies that better illuminate the epistemological importance of fire and other nonhuman agents in the landscape to Indigenous peoples in the boreal. This literature also documents the forced separation of Indigenous peoples from traditional cultural burning practices, a particularly harmful form of cultural severance, as well as more recent examples of Indigenous fire knowledge resurgence.

## Results

### Fire As an Agent, More Than a Tool

As alluded to early on, the existing literature suggests that historically, Indigenous peoples understood that humans were not the only agents of change in the boreal forest [70]. For Cree people for example, fire is seen as a being that has a spirit. Offerings (like tobacco or sage) are made to the fire spirit in ceremony (Phillip Campiou, Cree Elder, personal communication).^5^ Baker [72] has documented a creation story from Bigstone Cree Nation Elder Albert Yellowknee when she asked about fire use in the boreal forest: “… the creator breathes fire into two poplar trees for them to become humans. In this sense, fire is a life-giving force. He reminded me that everything is interconnected, fire included.” For many, this understanding of sacred fire persists. More than simply a form of combustion, landscape fires are understood as being connected to a wider set of human-land relationships and, in some cases, agents of change with profound implications for those that interact with it.

Anishinaabe of Pikangikum First Nation Elders, located in what is now northwestern Ontario, described fire in relation to a larger cosmological reality, conferring agency to beings like *beenaysee eshkotay* or thunderbirds, and the process of burning itself. Miller and Davidson-Hunt [47] explored how Elders perceived forest fires as beings “which [possess] agency and who intentionally create order in landscapes.” Elders also discussed fire as an expression of agency, a process capable of growth, travel, and both a source of destruction and renewal. Resting at night and active in the day, fire is understood as a living component of the landscape. While fire destroys and takes life, it is also a source of life. Burned areas are rapidly recolonized by plants and animals and provide new growth and increased food opportunity for both humans and relations [47], and have other impacts on forest renewal.

For Shoal Lake Anishnaabe, as described by Berkes and Davidson-Hunt [9: p. 42]:

*In the Anishinaabe perspective, the Creator placed the people in Iskatewizaagegan (Shoal Lake) and provided everything that they would need for their survival in that place. In return, the Anishinaabe hold the responsibility to maintain these gifts. Practices that harm these gifts can lead to consequences for an individual or the individual’s family. At the landscape scale, there is a basic duty upon the Anishinaabe not to influence abundance or distribution of habitats. In a workshop with elders in Pikangikum, the same principle emerged and was concisely translated into English as, “as was, as is”. The creation of blueberry patches through repeated burning was not seen as a contradiction of this principle. Burning or other disturbance simply reveals the different combinations of plants that are naturally present in the landscape*.

Further east in Labrador, for example, fire also has an important role for the Innu in their cultural life, being the center of many ceremonies [41•].

For Indigenous peoples in the boreal forest, fire is part of a complex network of relationships beyond that of just humans and fire. Fire is connected to a wide range of species on which Indigenous communities depend on, and the presence and absence of fire narrates how these relationships between humans, plants, and animals transpire. This is similar to how some other nonhuman entities such as glaciers, rivers, plants, and wildlife are understood as active agents and beings in the world [73, 74]. As such, several Indigenous scholars have described relationships between human and nonhuman beings in terms of treaties, care, and kinship [75, 76]. In some instances, fire is an important component of strengthening these relationships [77•]. Instead of conceiving fire exclusively as a tool, Indigenous peoples see fire, humans, and other elements of the environment as active components in the boreal, and link their epistemological worldviews to the relations between human and nonhuman entities on the land. Indigenous conceptualizations of fire, relation and land offer radical alternatives to dominant approaches to fire and the environment. The boreal needs fire [9], and people need the boreal.

### Fire Practices

The pre-settlement landscape in Canada was strongly influenced by Indigenous land management to enhance productivity, with fire providing the strongest means of landscape manipulation. The first research studies to document Indigenous fire practices in the boreal were early anthropological work that focused on fire as a tool. Lutz [78] confirmed that fire was used for campfires, signaling as a form of communication, for aiding in the application of spruce gum to repair birchbark canoes, to open dense understories to improve chances of a clear shot when hunting, to force game, including birds, to expose themselves as they moved away from the advancing fire front, for use in warfare, and a multitude of other reasons.

**Fig. 2:**
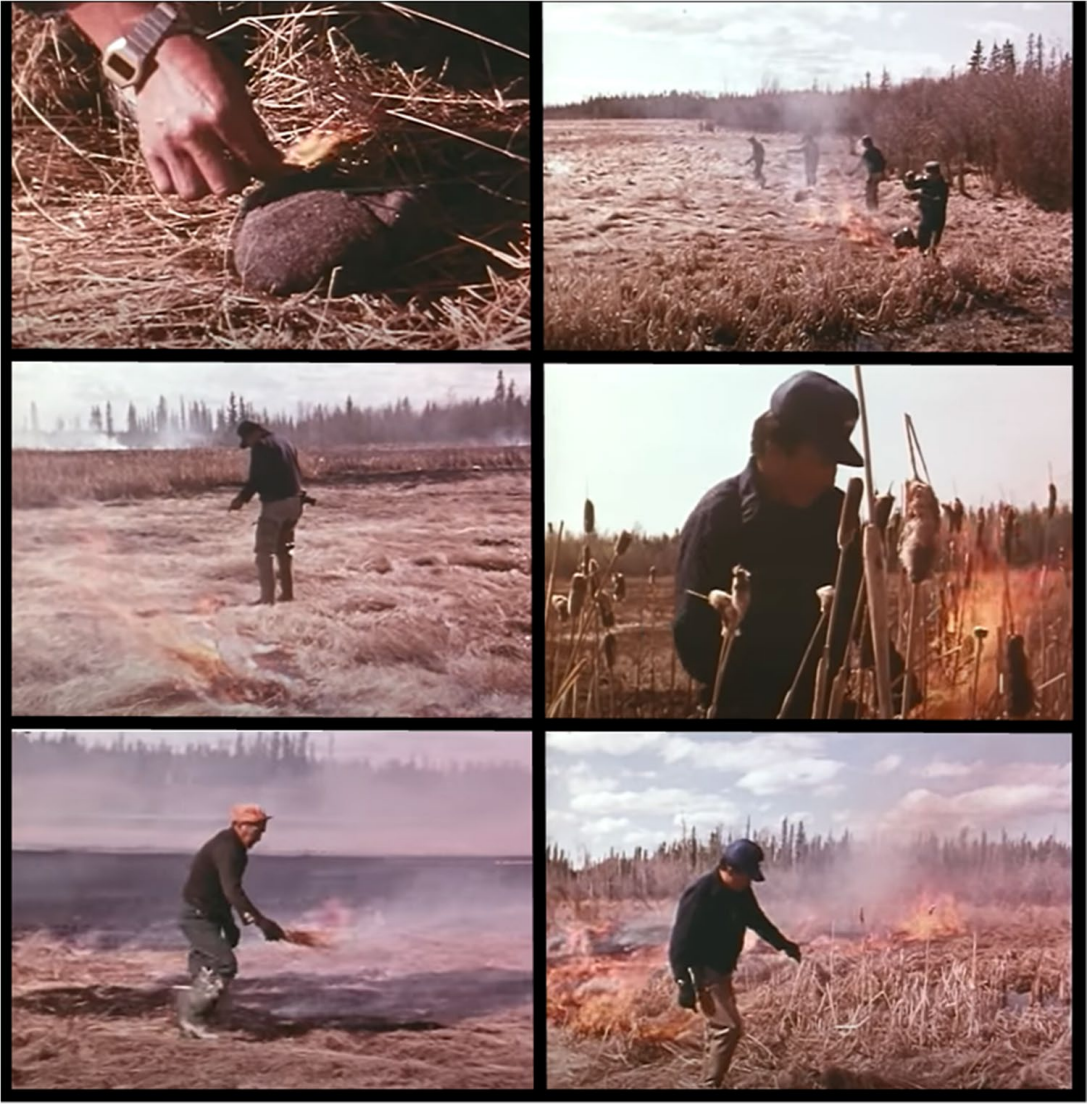
Still images from the film “Fires of Spring” featuring Dene and Woodlands Cree burners in northern Alberta [65]

The most extensive research program was conducted by Henry Lewis and Theresa Ferguson in the 1970s on fire use by the Dene and Woodland Cree in northern Alberta [36, 79–87]. This includes the seminal book “A Time For Burning” [84] and a 32.5 min documentary film entitled the “Fires of Spring” [80, 87] (Fig. 2). Their work focused on understanding the use of fire as a practice and of fire as a tool in achieving certain ecological conditions. They used archival and ethnographic research methods, including interviews with Elders, to document fire practices. The most important contributions of their research has been the extensive list of “reasons for burning” and documentation of mosaic patterns created through burning in the boreal forest [36, 79–82, 84, 85]. These included the maintenance of meadows, opening up grasslands, burning deadwood, extending the growing season, obtaining firewood, improving settlements and campsite areas, making and maintaining trails, opening up animal habitat, increasing berry production, reducing pests, religious reasons, and esthetic benefits. The Dene had a specific word for recently burned areas, *go-ley-dey*, and would share information about where they were and when hunting would be good in those areas [36]. Although the study did not focus directly on wildfire mitigation, Lewis and Ferguson [79–82, 84] found that Indigenous peoples wanted to use traditional burning practices around their communities in the spring to mitigate future wildfire risk in the more dangerous hot and dry months.

Lewis [84] also detailed techniques for burning and associated knowledge that was required to achieve desired cultural objectives. He found Indigenous peoples he spoke with were highly knowledgeable about seasonality, timing, fuel conditions, relative humidity, wind, other general weather conditions, slope steepness, and natural fire breaks as they related to burning. They spoke about the frequency and severity of fire needed to achieve specific burning outcomes, which was dependent on what they wanted to achieve. For example, meadow burning was best done in the spring (with snow still in the forest edge) through low intensity burns on a frequent rotation (i.e., every few years) [84]. Such detailed fire knowledge dispels myths of the “careless Native,” who would have left campfires unattended and undertook other careless activities involving fire. Rather:

*In some cases, where grasses had not sufficiently dried, Indians would set and leave campfires with one or more smoldering logs extending into the grasses; these delayed fuses would later ignite the area, sometimes days after their departure. As one Indian trapper noted: ‘When we’d come off the trapline it might be too wet to burn the sloughs and creeks. So we’d just build a big campfire and leave it. Maybe couple weeks later, when the grass is really dry, the grasses would all get burned up, but the fire wouldn’t go anywhere because it was still too damp in the bush*. [84]

Ferguson [88] completed later work in 1985 and 1986 with Cree-Chipewyan peoples in Wood Buffalo National Park (Northern Alberta). Participants notes that burning was primarily conducted around settlement areas, primarily to “clean-up,” provide hay for livestock, and prevent deciduous tree encroachment into meadows. Interestingly, a main purpose of burning certain campsites in the spring was to discourage snakes. Ferguson [88] had several hypotheses for why there was less knowledge about fire and burning done in this area compared to others, including the following: (a) people are concealing information from the research team due to concerns about government fire policy, (b) soil salinization in certain areas created and maintained natural open areas, (c) Chipewyan participants in the study were recent “immigrants” to the area, and (d) early fire suppression activities in this area meant that there is little memory of burning practices.

In Labrador, fire was used by Indigenous peoples to burn berry patches, improve bear hunting, create habitat biodiversity (including using small fires to create early succession areas for caribou), open travel routes, create stands of deadwood, change migration patterns and herd movements of caribou, and signaling [41•]. Fishing with fire was also common in Innu communities, where salmon would be attracted with birchbark torches before spearing [41•]. This practice continues to contemporary times, when fire is also used to control grass mouse populations, reduce traveling times for firewood, improve or create berry patches, and promote early succession trees like poplar that carvers use [41•]. Sod and rotten wood is also used in smokehouses or as insect repellent [41•].

In Northeastern British Columbia, the Dene and Cree of Fort Nelson First Nation used fire for multiple reasons, including the following: grass burning and clearing, vegetation regrowth, esthetics, spiritual/ceremonial, hunting, protection from animals and insects, warmth and cooking, communication, and light [89]. Spring burns were more common than fall burns due to the importance of snow being used as firebreaks, and an activity the whole family would engage in together. Burns are still employed to improve bison habitat in their territory [89].

In Ontario, Indigenous peoples used fire to promote early succession forests that better met their needs [90]. For Pikangikum First Nation, it is understood fire can have the potential to destroy life, but also be a source of life. Burned areas rapidly attract new plants and animals and provide new growth and increased food opportunity [37, 38], and have other influences on forest renewal. Shoal Lake Anishnaabe frequently used fire to create disturbances in the forest canopy, to maintain habitats in early stages of succession, until the practice was banned; they also used fire to establish gardens or blueberry patches using different techniques depending on the site and objective, as well as to control understory vegetation, enhance hunting visibility, and to keep campsites free of brush [9, 46]. At Peavine Métis Settlement in what is now Northwestern Alberta, fire was used to “clean” the land and later to promote agricultural and subsistence practices, including positive effects on berry production and hunting activities [91]. In many cases, cultural burning had alternative impacts, including wildfire risk reduction through burning of fuels adjacent or within the community, including in Northern Saskatchewan in burning done by Swampy Cree and Métis peoples [92]. Participants noted that intentional fires sometimes got away, but they tended to be small and easy to put out. In Bigstone Cree Nation and Fort McKay First Nation, fire was particularly important to promote berry patches and medicines, reduce mosquitoes and black flies, and keep smaller waterways and trails open: bears, berries, and fire were also noted as having an important relationship [72].

Further North in what is now Alaska, research conducted by Natcher and co-authors [93] found that two different Athabascan groups had very different perceptions of wildfire and cultural burning. The Gwich’in used cultural burning to clear underbrush, improve habitat, and aid in locating and pursuing game, for example, using fire to kill standing timber to create fences to influence caribou movement. They also used fire to signal one another, create dry firewood, and combat insects. While the Gwich’in of Alaska used fire strategically as part of their land management, neighboring Koyukon viewed fire more as disadvantageous to territorial use and do not seem to have recollection or history of using fire volountarily [93]. As Natcher and co-authors [93] hypothesized: “Some of the factors that have contributed to this regional and cultural variability may include differences in the terrain between the Gwich’in and Koyukon territories, lightning-strike density and the occurrence of natural disturbance, and differences in subsistence and settlement patterns. Together, these factors offer some explanation for why the Gwich’in and not the Koyukon used fire to modify the landscape.”

**Fig. 3:**
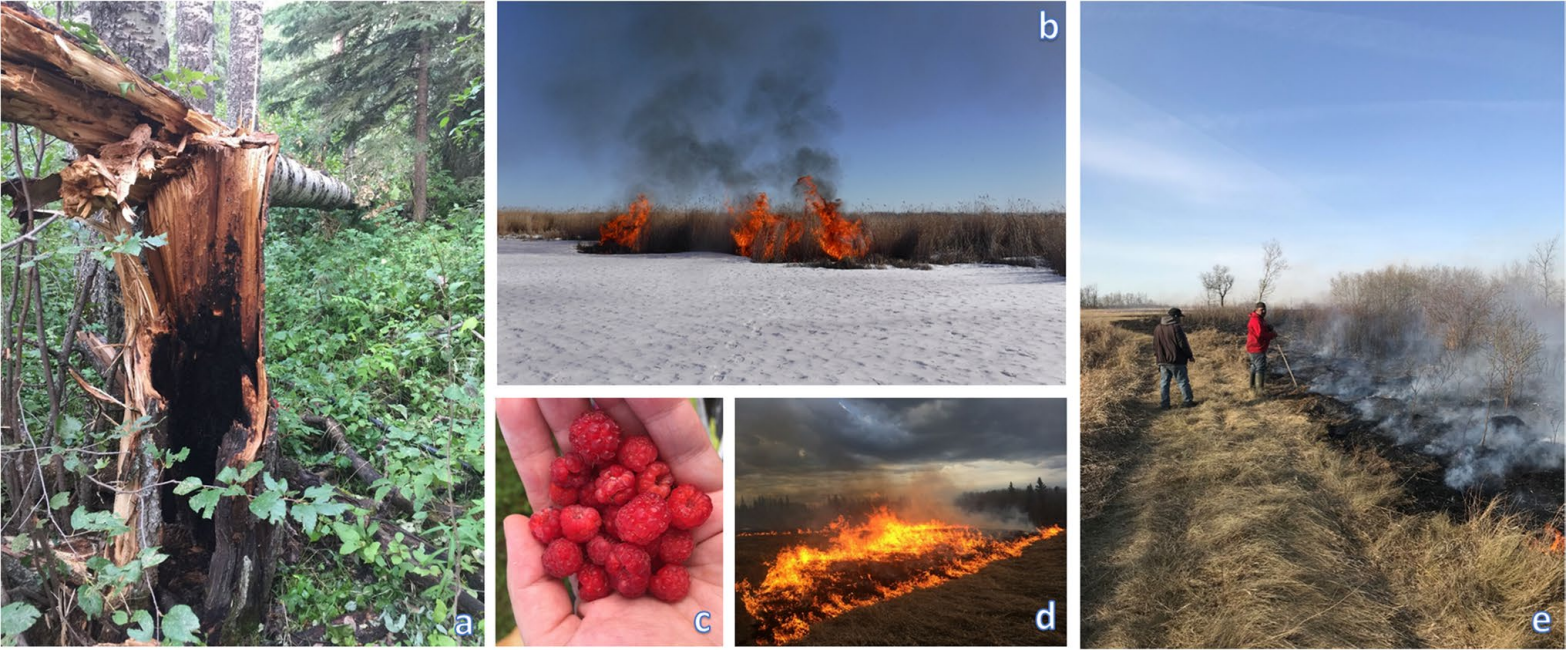
Some scenes related to Indigenous fire in the boreal forest. **a** Lightning strike on a trembling aspen tree in area that had been culturally burned (Amy Cardinal Christianson). **b** Burning in winter by Cumberland House Métis to improve muskrat habitat (Renee and Solomon Carriere). **c** Wild raspberries a few years after a cultural burn (Amy Cardinal Christianson). **d** Burn on Whitefish Lake First Nation 459, Atikameg reserve (Paul Courtoreille). **e** Peepeekisis First Nation burn (Michelle Vandevord)

Burning still has many current implications, for fishing, hunting, and cooking of wild game. As a tool, fire on the landscape is a key disturbance that contributes to favorable conditions for Indigenous peoples [94] (Fig. 3). For example, in Pikangikum, although many cultural burning practices have become reduced in both size and frequency, this activity is still seen as a duty of Anishinaabe people, as they feel a custodial duty to the land [37, 38].

### The Role of Wild Fire

The boreal biome’s many ecosystems are continuously transformed by lightning-ignited fires that travel across landscapes, with profound implications for human and nonhuman beings alike. The resulting mosaic of forest and wetland communities are a major driver of renewal and opportunity. While most research on human-fire relationships is preoccupied with the Indigenous *uses* of fire, it is important to note that all fires in the so-called boreal are entangled with human relationships, even those perceived to be *wild* and without consequence for human actors.

A common theme in the literature is the emphasis put on the use of areas that have experienced such events and the distinct role — both positive and negative — these wildfires play in the creation of Indigenous cultural landscapes [28••]. For instance, the Dene and Woodland Cree accessed areas affected by wildfire to collect firewood in the otherwise impenetrable boreal forest [84]. They also made use of berry patches from wildfires [84]. In many communities, these are both historical and contemporary practices. However, the summer wildfires were/are less desirable than cultural burning because of the associated danger and their uncontrolled nature (e.g., disruption of trails, traplines) [85].

The Koyukon also noted differences between low/moderate intensity wildfires, which occurred earlier in the season, and high intensity wildfires that occurred later in the season and tended to have more damaging effects on permafrost sites [95]. They mentioned the positive effects of wildfire on berries and moose populations, but negative effects including “the loss of important places, difficulties trapping, caribou displacement, and the deaths of small animals” [95: p. 37]. Koyukon knowledge keepers noted differences in wildfire severity and resultant effects in mature, spruce-dominated stands versus non-spruce brushy areas, berry patches, and lichen-dominated areas [95]. Thus, relationships to burned areas can be diverse and are not universal in nature across the boreal.

Landscapes impacted by wildfires effect a number of species on which Indigenous Nations depend on. Of note is the role lightning-ignited fires have played in the transformation of landscapes, and associated interactions among Indigenous peoples, woodland caribou, deer, bison, moose and other fur-bearing and other game species [96, 97]. However, the effects of wildfire on flora and in turn fauna, such as the creation of habitat, is beyond the scope of this paper, as noted previously in the methodology.

### Fire and Cultural Keystone Species

Wildlife species in the boreal forest are well adapted to fire of varying frequency, size and severity [21, 23, 98]. While there is a paucity of *western* research on the short- and long-term responses to cultural burning, numerous studies have shown that naturally occurring fires promote plant and animal diversity by creating opportunities for tree regeneration [99]; establishing habitat conditions for shade-intolerant plants [100]; and modifying the spatial and temporal availability of resources such as light, water and nutrients and contributing standing snags and other coarse woody debris that are used by a plethora of invertebrates, fungi, mammals, birds, and other wildlife after disturbance [101]. Among the plants and animals that benefit from fire are Indigenous *cultural keystone species* (CKS) “that shape in a major way the cultural identity of a people, as reflected in the fundamental roles these species have in diet, materials, medicine, and/or spiritual practices” [102]. They include moose, bison, and numerous berry-producing shrubs, including wild huckleberry and a variety of blueberry species that are important as both food and medicinal plants [9]. Indigenous peoples in the boreal have used fire to promote the populations of these and other CKS in a myriad of different ways. They include Anishinaabe burning of aspen parkland to expand prairie habitat for bison, Dunne_zaa burning along creeks and sloughs to improve forage and attract fur-bearing and game species (e.g., mink, weasel, marten, lynx and even wolves) to traplines [36] and Gitksan and Wet’suwet’en landscape burning to create and maintain productive berry fields under complex systems of fire management and shared governance [103].

The CKS concept has evolved in recent years, with a recognition of the broader biocultural significance of fire as a *cultural keystone practice* by Indigenous Nations living in the boreal, and elsewhere, in food production, plant cultivation, and for other traditional resource purposes. Deur and Turner [104] draw attention to the use of fire, as an Indigenous technology for plant cultivation, in the translation of the Kwak’wala word *q’waq’wala7owkw*, or *keeping it living*, that was shared with them by Hereditary Chief Kwaksistala Adam Dick.^6^ The phrase has a number of associations, most notably that Indigenous Peoples use fire and other traditional practices to modify CKS, including plants and their habitats, frequently and purposefully in order to keep those valued biocultural attributes in their territories alive and flourishing on the landscape [105].

There are numerous examples in the literature of how Indigenous peoples of the boreal have used fire in a manner that is consistent with *q’waq’wala7owkw* (keeping it living) and other analogous Indigenous land management systems to promote the extent and productivity of cultural keystone plants, most notably berry-producing species which re-sprout vigorously from below-ground stems and produce abundant crops of fruit with periodic burning [9, 41•, 72, 84, 89, 103, 105–109]. As Pat Namox was to note (quoted by Antonia Mills, no date: p. 156; cited in Gottesfeld [103]):

... *When it is the right time he [the chief] burns the berry patches so the berries are fat and plump. If he didn't do that the berry patches would become old and overgrown and there would be berries but they would just be small. But he knows when to burn so that it cleans up just the berry patch and doesn't spread to the trees*....

Indigenous management of berries demonstrates how, contrary to the prevailing description of “hunter-gatherer,” Indigenous Peoples actively managed and cultivated plant species in the boreal and other regions to foster the abundance and quality of preferred wildlife species. Indeed, the management of berry patches was guided by complex political and social systems which varied by group, but which served to conserve and enhance the yield of favorable plants [110, 111]. For example, burning for berries by Dene in the Meander River area of what is now northwestern Alberta was done by women, given their role in the community as berry harvesters [84].

References to the management of plant and animal species in the boreal with cultural burning are documented in the literature. We present over three dozen species in Table 1, ranging from vascular plants such as blueberry, to insects such as mosquitoes and other biting flies, to numerous fur-bearing and game species, ranging from smaller mammals (e.g., rabbit) to large ungulates, like moose and caribou that were managed by Indigenous Peoples using fire.

**Table 1:** Species that are known to have been managed with Indigenous burning practices in the boreal forest

Common name	Species	Indigenous Nation	Indigenous name	Geographical location	Reasons for burning	Key references
Lowbush blueberry; Dwarf blueberry	*Vaccinium angustifolium; V. caespitosum*	Anishnaabe; Gitksan and Wet’suwet’en	yintimï? (W) ‘myahl(G) miinan (A)	Northwestern Ontario; Northwestern Alberta, Northwestern British Columbia	Creation, maintenance and renewal of productive berry patches, including enhanced berry patch productivity (number of berries), increased berry sweetness, increased size of berries	[9, 72, 106, 110]
Velvet-leaved blueberry; Oval-leaved blueberry	*Vaccinium myrtilloides; V. ovalifolium;*	Anishnaabe; Gitksan and Wet’suwet’en	dïndze (W) miinan (A)	Northwestern Ontario; Northwestern British Columbia	Creation, maintenance and renewal of productive berry patches, including enhanced berry patch productivity (number of berries), increased berry sweetness, increased size of berries	[9, 106, 110]
Black huckleberry	*Vaccinium membranaceum*	Gitksan and Wet’suwet’en; Nisga’a	digï (W) simmaa’y or sim’maa’y (G)	Northwestern British Columbia	Creation, maintenance and renewal of productive berry patches, including enhanced berry patch productivity (number of berries), increased berry sweetness, increased size of berries, control of pests	[105–108, 110]
Soapberry	*Shepherdia canadensis*	Gitksan and Wet’suwet’en	niwis (W)	Northwestern British Columbia	Creation, maintenance and renewal of productive berry patches	[110]
Bilberry	*Vaccinium uliginosum L., V. boreale*	Innu	nissiminanakashi (I)	Labrador	Increased berry production	[41•]
Mountain cranberry; Lingonberry	*Vaccinium vitis-idaea L*	Innu	uishatshimin(an)akashi (I)	Labrador	Increased berry production	[41•]
Wild strawberry	*Fragaria vesca*	Dene; Cree	įdziáz (D) otêhimin (C)	Northeastern British Columbia	Increased berry production	[89]
Saskatoon serviceberry	*Amelanchier alnifolia*	Dene; Cree	k’įnjíe (D) misâskwatômin (C)	Northeastern British Columbia	Increased berry production	[89]
Raspberry	*Rubus idaeus*	Dene; Cree	dakáłjíe (D) ayôskan (C)	Northeastern British Columbia	Increased berry production	[89]
Riceroot	*Fritillaria camschatcensis*	Gitksan and Wet’suwet’en	c’inkalh (W)	Northwestern British Columbia	Possible burning of floodplain garden sites to enhance bulb production	[109, 110]
Meadow management		Multiple Nations		Throughout boreal	Aspen and birch “overgrowth/expansion” into meadows managed through fire. Aspen and white spruce stands were the best for “converting” to meadows because of underlying soils	[9, 36, 38, 41•, 82, 89, 91]
Grass	*Calamagrostis canadensis*	Innu	mashkushi (I)	Labrador	Promote new grass growth, control grass mouse populations	[41•]
Willow	*Salix* spp.	Multiple Nations	k’ai (D)	Northern Alberta, Northeastern British Columbia	Control spread, especially for streamside trails. Attract ungulates to new growth	[84, 89]
Poplar	*Populus* spp.	Innu	mashi-mitush (I)	Labrador	Promotes early succession trees important for carvers	[41•]
Mosquitoes, black flies and other biting insects		Multiple Nations	dejúli (D) T̲s̲’iih (DZ) shatshimeu (I)	Northern Alberta	Controlled by creation of open areas through burning	[84]
Muskrat	*Ondatra zibethicus*	Dane_zaa	dlechuk (DZ)	Northwestern Alberta	Stimulate new root growth as a source of food in marshes, sloughs and along lakeshores	[36]
Plains Bison	*Bison bison bison*	Anishnaabe	iiníí (B)	Aspen parkland regions of the Prairie Provinces	Creation of productive forage habitat	[46]
Woodland Bison	*Bison bison athabascae*	Multiple Nations	nįnteliįjeré (D)	Northern Alberta (in particular Wood Buffalo), Northeastern British Columbia	Open migration paths to bring bison north, promotion of “buffalo grasses.”	[84, 89]
Moose	*Alces alces*	Dane_zaa; Dene, Cree	deníí (D) hadaatseʔ (DZ)	Northwestern Alberta, Northeastern British Columbia	Stimulate new shoot growth as a source of food; creation of productive forage habitat; attract animals to hunting areas	[36, 89]
Elk	*Cervus canadensis*	Dene. Cree	dzendhił (D) wâwâskesiw (C)	Northeastern British Columbia	Stimulate new shoot growth as a source of food; creation of productive forage habitat	[89]
Horse (wild and domesticated)	*Equus ferus caballus*	Dane_zaa, Dene, Cree	tlęchuk (DZ) łįchok (D) mistatim (C)	Northwestern Alberta. British Columbia	Production of forage	[36, 89, 105]
Mink	*Vison vison*	Multiple Nations	Ębaa (DZ)	Northwestern Alberta	Attract animals to traplines	[36, 84]
Weasel	*Mustela spp.*	Multiple Nations	taadle (DZ)	Northwestern Alberta	Attract animals to traplines	[36, 84]
Fisher	*Pekania pennanti*	Multiple Nations	nǫhgaashe (DZ)	Northwestern Alberta	Attract animals to traplines	[84]
Marten	*Martes americana*	Multiple Nations	thah (D) Uuschęą (DZ)	Northwestern Alberta	Attract animals to traplines	[36, 84]
Lynx	*Lynx canadensis*	Multiple Nations	nǫdaa (DZ)	Northwestern Alberta	Attract animals to traplines	[36, 84]
Beaver	*Castor canadensis*	Multiple Nations	tsá (D) tsaaʔ (DZ)	Northern Alberta	Stimulate new shoot growth as a source of food in marshes, sloughs and along lakeshores	[36, 84]
Fox	*Vulpes vulpes*	Multiple Nations	nąghídhe (D) yuus̲e (DZ)	Northern Alberta	Attract animals to traplines	[36, 84]
Ptarmigan	*Lagopus* spp.	Innu	uapineu (I)	Labrador	Create habitat	[41•]
Rabbit	*Lepus* spp.	Multiple Nations	gah (D) Gaah (DZ)	Northern Alberta	Stimulate new shoot growth as a source of food	[36, 84, 89]
Mouse	*Mus* spp.	Dene	dlúne (D) Dlwęą (DZ)	Northern Alberta	Stimulate new shoot growth as a source of food	[36]
Wolf	*Canis lupus*	Dane_zaa	ch’ǫneʔ (DZ)	Northern Alberta	Attract animals to traplines	[36]
Deer	*Odocoileus* spp.	Multiple Nations	yáhtųe (D) yaatune (DZ)	Northern Alberta, Northeastern British Columbia	Stimulate new shoot growth as a source of food; creation of productive forage habitat; attract animals to hunting areas	[36, 84, 89]
Waterfowl (variety of species, including ducks)	*Species belonging to the family Anatidae*	Multiple Nations	Ghaje (DZ) t̲s̲it̲s̲ (DZ)	Northern Alberta	Stimulate new root growth as a source of food in marshes, sloughs and along lakeshores	[36, 84]
Birds	*Aves*	Dene. Cree		Northeastern British Columbia	Create habitat	[89]
Woodland Caribou	*Rangifer tarandus*	Tahltan, Innu	hodzih (T) madziih (DZ) atik^u^ (I)	Northwestern British Columbia, Labrador	Approach animals downwind under cover of smoke, promote small patches of early succession forest, change migration patterns, hunting	[41•, 105]
Barren-ground Caribou	*Rangifer tarandus groenlandicus*	No Nation specified		Southern limits of the barren-ground caribou winter range	Stimulate the growth of lichens and other forest plants through removal of byrophytes (liverworts, hornworts and mosses) in upland forest and muskeg	[84]
Bear	*Ursidae*	Multiple Nations	sas (DZ) or dleye (DZ), dlézi (D)	Northern Alberta, Northeastern British Columbia, Labrador	Attracted to new berry growth	[41•, 72, 74, 84, 89]
Fish	*Various species*	Innu		Labrador	Use bonfires or torches to attract fish	[41•]
Garter snake	*Thamnophis sirtalis*	Chipewyan		Northern Alberta	Burned off grass in fishing camps to discourage snakes	[88]

(A): species name in Anishinaabemowin. (B): species name in Blackfoot. (C): species name in Cree. (D): species name in Dene. (DZ): species name in Dane_zaa Záágé. (G): species name in Gitksan. (I): species name in Innu. (T): species name in Tahltan. (W): species name in Wet’suwet’en

### A Fire History of Colonialism, Cultural Severance, and Increased Vulnerability

Historically, settler governments criminalized Indigenous fire practices, thereby disrupting the land use practices of Indigenous peoples in the boreal [11, 35, 36, 62••]. These actions have led to long-term impacts for both Indigenous Nations and the boreal landscape that are felt to this day. The suppression of fire as a traditional and customary management practice can be thought of as *cultural severance*, first coined by Rotherham [112], defined as an act, intentional or not, that functionally disrupts relationships between people and the land. The impacts of cultural severance are diverse. They include the loss of valued biocultural components, broad successional shifts in landscapes, possible declines in unique biodiversity (e.g., pyrospecies), declines in wildlife populations and culturally significant plants, and even facilitating the spread of invasive species [113]. In the specific context of anthropogenic fire, the impacts of cultural severance can also increase vulnerability to catastrophic fires because of increases in fuel build-up and continuity [6, 11, 29, 114, 115].

Fire bans on cultural burning implemented by settler administrations were consistent with a wider strategy that aimed to criminalize relationships between Indigenous Nations, land, and each other. Examples of other strategies included the criminalization of cultural burning, the control of travel onto the land, forced sedentarization and displacement from their territories [116, 117], the establishment and maintenance of the reserve system, and residential schools [118]. The impact of assimilation efforts on Indigenous Nations has been to disrupt the transmission of Indigenous knowledge. Further, fire prevention policies in Canada [35] and Alaska [119•] have reduced the use of fire by Indigenous peoples, although some still burn in their territories [37, 41•, 77•, 89, 91]. These impacts also varied by Nation and geographic region, with different periods of trade and settlement leading to a varied colonial experience. As the occupation by European settlers started earlier in the East, it suggests more eastern Nations experienced a longer period of disruption from their cultural practices compared to western Nations.

In Newfoundland, for example, fire bans began as early as 1610 when the governor enforced authority over “crown” timber [120]. Similar fire bans followed, with British Columbia establishing a fire ban, accompanying fines and imprisonment via the province’s Bush Fire Act of 1874 [121, 122]. In the 1920s, Indigenous people in Manitoba were arrested for starting intentional fires [123]. In the 1970s, Indigenous peoples in Northern Alberta still wanted to use traditional burning practices around their communities in the spring to mitigate future wildfire risk in the more dangerous summer months; however, this practice was illegal due to government fire prevention policy [84]. In present-day Alberta, Métis reported that burning of the forest did not occur frequently following the enactment of provincial prevention policies and burning was discouraged by the community because of agency-imposed fear of an out-of-control wildfire, fines, or imprisonment [91]. In present-day Ontario, Elders also describe being imprisoned for the duration of the fire season for intentionally setting fires [37]. Colonialism and fire exclusion followed a similar path to that in California, where wildfire management agencies racialized “light burning” and delegitimized or erased Indigenous peoples and knowledge through three key narratives: (1) Discrediting- “savage” narrative; (2) Downplaying-vanishing “Indian” narrative; and (3) Erasure-*terra nulius* narrative [124].

Although provincial, territorial, and federal fire management agencies in recent decades have shown a greater acceptance for using fire on the landscape, agency-certified burners with “western” prescribed fire techniques predominate, such as the use of accelerants and production burning techniques. Discouragement of intentional burning by non-agency burners has resulted in Indigenous knowledge not being passed on to younger generations [77•, 91, 95, 125]. Pikangikum Elders in Northwestern Ontario expressed the view that theirs was the last generation with the knowledge of fire use and that they wished to pass it on to younger generations before this collective knowledge vanished [37]. Further research should be carried out to describe how Indigenous burning was externally policed, how and why it was resisted, and what barriers to Indigenous fire practices still remain. Answers to these questions will be key to facilitating change, documenting past practices by Indigenous Nations and colonial administrations, and supporting the revival of such Indigenous practices.

Although cultural burning practices are now reduced in size and frequency across the North American subcontinent, fire knowledge still exists in Indigenous communities throughout the boreal region. For instance, the activity is described as a duty by Anishinaabe people of Pikangikum First Nation, as they feel a custodial duty to the land [47]. This is echoed by a number of Indigenous Nations [1•, 11, 77•]. In many instances, Nations have made efforts to renew human-fire relations despite their relentless interruption by ongoing colonialism. Such efforts to revitalize cultural burning have made direct reference to the Truth and Reconciliation Commission’s Calls to Action and Sect. 34 of the United Nations Declaration on the Rights of Indigenous Peoples [118, 126, 127].

Settler governments have also excluded First Nations from decision making about fire management policies [27, 28••, 37, 62••]. Many fire management agencies have now come to accept fire is a natural, needed process in many forests, but have taken an extreme stance in some areas that have major impacts on Indigenous peoples and cultural landscapes. For instance, strategic planning about allowing large fires north of the 51st parallel neglected to consult local First Nations [37]. In Alaska, the Koyukuk and Northern Unit Innoko National Wildlife Refuge Fire management plan “failed to acknowledge th[e] variability [in wildfire reported by Indigenous peoples] and highlighted only the potentially desirable effects” [95]. However, these wildfires have been devastating to traditional territories [28••]. Management decisions defining where fire suppression efforts occur has reflected a lack of recognition of the existence and values of Indigenous Nations [35]. In Northern Saskatchewan, for example, Indigenous groups did not consent to “Let-it-Burn” fire policies, where suppression was not conducted if government-determined “values” were not at risk [28••]. Even for Indigenous peoples who may not currently practice local fire knowledge, given the fact that fire management impacts their territories, greater consideration and actions must be taken to involve Indigenous peoples and their perspectives in future fire management decision making.

Contemporary approaches to fire and forest management in Canada remain nested in approaches that position forests and land as timber and other resources to be protected from fire and consumed in the global marketplace [128, 129]. Indeed, colonialism has facilitated a shift in land ownership, jurisdiction (and thus access to land) and has rendered diverse ecosystems as *resources* [130]. This has led some scholars to question a number of the tools and concepts that embrace a “values-at-risk” approach to fire management. All fire management agencies list human life as the first value to protect. After this, value rankings can vary and often focus on structures, infrastructure, and industry. These tools are embedded in capitalist understandings of value that position resources as an asset and negate alternative approaches to valuing and caring for the landscape [131–133]. Only select agencies have begun the work to identify Indigenous sites in their inventory of values-at-risk. For some Indigenous scholars writing in the context of Canada, colonialism and capitalism are one in the same, whereby the displacement of Indigenous Nations made way for a variety of capitalist ventures at the cost of Indigenous Peoples and the land [134]. In ordering relationships between landscapes and people in this way, governments have made Indigenous Nations more vulnerable [17, 26, 135, 136].

## Conclusions

Indigenous knowledge systems have allowed Nations to survive for thousands of years in a constantly changing world [55•]. Indigenous peoples in the boreal have applied fire on their landscapes for a multitude of reasons. They understand fire as an active, alive agent. As an agent, fire is capable of movement, destruction, and creation, acting on the landscape to create order, within a living, connected environment. Fire operates on the landscape, co-existing with and challenging people of the boreal forest.

This paper summarizes a diverse body of scholarly literature documenting Indigenous perspectives and interactions with fire on the landscape. This body of research “collectively refute[s] the idea that… forests are essentially unchanged by people, either in the past or present day” [41•: p. 11]. This paper challenges the dominant narrative of wildland fire history in the boreal forest that has to date focused on large-scale fires and has limited engagement with small-scale fires that often escape the detection of large-scale measurements. Factoring in small-scale burning, including Indigenous historical accounts, allows for a more holistic and accurate depiction of the place of fire in the boreal. As discussed earlier, this paper also challenges the dominant narrative that western biophysical research is the primary way of knowing. Indigenous knowledges are presented as distinct, holistic, and robust modes of knowing land and fire that have been millenia in the making. We call on our non-Indigenous colleagues who research on and write about the boreal forest, to include Indigenous peoples and perspectives in their work — not as footnotes or in the acknowledgement sections, but as equal peers and collaborators.

Due to climate and forest fuel changes, Indigenous communities are at increased risk of evacuations and wildfire related impacts [29, 62••]. There is increasing interest by government agencies and non-Indigenous researchers to “integrate” or “incorporate” Indigenous knowledge about fire, including cultural burning practices, into colonial management systems [138]. This enthusiasm to engage Indigenous knowledge about fire must also include discussions regarding Indigenous leadership and engagement in forest and wildfire management decisions, including training, certification, and liability issues. Indigenous peoples should not only be informing decision-makers. There needs to be a shift in power so that they are the ones making the decisions about their own territories.

## Supplementary Information

Below is the link to the electronic supplementary material.

Supplementary file1 (PDF 143 KB)

Supplementary file2 (PDF 129 KB)

Supplementary file3 (PDF 148 KB)

Supplementary file4 (PDF 143 KB)

Supplementary file5 (PDF 177 KB)

Supplementary file6 (PDF 151 KB)

## Footnotes

1 The majority of Indigenous communities in the boreal region are First Nations and Métis.

2 For an overview of state-recognized Indigenous lands, Indigenous territories, and their connection to intact ecological areas of Canada, see [11].

3 See Feit [66: p. 69], for example, who attributes all fires in the boreal territory of the “Mistassini Indians” (general geographical area) as lightning caused and states “In the boreal forest region, while aboriginal man undoubtedly has had a considerable effect, natural causes, namely lightning, provide an adequate explanation and appear to have been at work prior to the presence of man”. Feit [66: p.21] also writes “Low [67] attributed these fires to the Indians themselves who started them through carelessness or intentionally. Many nineteenth century explorers agreed with Low, as did the popular literature. They attributed the fires, the declines in game populations and the resultant starvation of the Indians to the carelessness, or worse the avarice, of the hunters and gatherers”. Bell [68] is yet another example of a historical account from a Western scientist who completely ignored Indigenous cultural practices and knowledge systems in the boreal with respect to fire use. Oberndorfer [41] also discusses the biases behind historical accounts of fires and Indigenous burning practices in Labrador, including a European settler pyrophobia and authors who had no experience with the benefits of prescribed fire in a boreal context.

4 This is not to say that Indigenous peoples do not have other ways of sharing fire knowledge outside the academy, including through non-traditional methods like workshops, camps, podcasts, newsletters, and blog posts. For example, the “Good Fire: Stories of Indigenous Fire Stewardship” podcast features Indigenous fire knowledge holders from around the world (https://yourforestpodcast.com/good-fire-podcast).

5 Although beyond the scope of this paper, the role fire plays in ceremony and spirituality more broadly, in the case of smudging, for example, should not be overlooked [70].

6 The term q’waq’wala7owkw is Kwak’wala spoken by Kwakwa̱ka̱'wakw on the east side of Vancouver Island and the adjacent mainland. Although not specific with respect to the boreal, it echoes the sentiment shared by other Indigenous Nations working with fire.
