# Supplementary material for: Centering Indigenous Voices: The Role of Fire in the Boreal Forest of North America
Source: Curr For Rep. 2022 Jul 27;8(3):257–76. doi: 10.1007/s40725-022-00168-9 (PMC9537118; doi:10.1007/s40725-022-00168-9)
Supplement: Supplementary file 1 — Supplementary file1 (PDF 143 KB) [file 40725_2022_168_MOESM1_ESM.pdf]

Disclosure of Potential Conflicts of Interest

Instructions

The purpose of this form is to provide readers of your manuscript with information about your other interests that could influence how they receive and understand your work. The form is designed to be completed and stored electronically. Each author should submit a separate form and is responsible for the accuracy and completeness of the submitted information. The form is in seven parts.

1. Identifying information.

1. Given Name (First Name): Amy

2. Surname (Last Name): Christianson

3. Date: June 17, 2021

4. Are you the corresponding author? ☑ Yes ☐ No

5. Manuscript Title: Historical Role of Fire in the Culture of Indigenous Peoples in Northern North Am

2. The work under consideration for publication.

This section asks for information about the work that you have submitted for publication. The time frame for this reporting is that of the work itself, from the initial conception and planning to the present. The requested information is about resources that you received, either directly or indirectly (via your institution), to enable you to complete the work. Checking "No" means that you did the work without receiving any financial support from any third party -- that is, the work was supported by funds from the same institution that pays your salary and that institution did not receive third-party funds with which to pay you. If you or your institution received funds from a third party to support the work, such as a government granting agency, charitable foundation or commercial sponsor, check "Yes".

Did you or your institution at any time receive payment or services from a third party (government, commercial, private foundation, etc.) for any aspect of the submitted work (including but not limited to grants, data monitoring board, study design, manuscript preparation, statistical analysis, etc.)?

Are there any relevant conflicts of interest? ☐ Yes ☑ No

If yes, please fill out the appropriate information below:

Name of Institution/Company	Grant?	Personal Fees?	Non-Financial Support?	Other?	Comments

3. Relevant financial activities outside the submitted work.

This section asks about your financial relationships with entities in the topic-specific arena that could be perceived to influence, or that give the appearance of potentially influencing, what you wrote in the submitted work. You should disclose interactions with ANY entity that could be considered broadly relevant to the work.

Report all sources of revenue paid (or promised to be paid) directly to you or your institution on your behalf over the 36 months prior to submission of the work. This should include all monies from sources with relevance to the submitted work, not just monies from the entity that sponsored the research. Please note that your interactions with the work's sponsor that are outside the submitted work should also be listed here. If there is any question, it is usually better to disclose a relationship than not to do so.

For grants you have received for work outside the submitted work, you should disclose support ONLY from entities that could be perceived to be affected financially by the published work, such as foundations supported by entities that could be perceived to have a financial stake in the outcome. Public funding sources, such as government agencies, charitable foundations or academic institutions, need not be disclosed.

Place a check in the appropriate boxes in the table to indicate whether you have financial relationships (regardless of amount of compensation) with entities as described in the instructions.

Are there any relevant conflicts of interest? ☐ Yes ☑ No

If yes, please fill out the appropriate information below:

Name of Institution/Company	Grant?	Personal Fees?	Non-Financial Support?	Other?	Comments

4. Intellectual Property.

This section asks about patents and copyrights, whether pending, issued, licensed and/or receiving royalties.

Do you have any patents, whether planned, pending or issued, broadly relevant to the work? Yes ☐ No ☑

5. Relationships not covered above.

Use this section to report other relationships or activities that readers could perceive to have influenced, or that give the appearance of potentially influencing, what you wrote in the submitted work.

Are there other relationships or activities that readers could perceive to have influenced, or that give the appearance of potentially influencing, what you wrote in the submitted work?

Yes, the following relationships/conditions/circumstances are present (explain below):

No other relationships/conditions/circumstances that present a potential conflict of interest

6. Human and Animal Rights.

Research involving human subjects, human material, or human data, must have been performed in accordance with the Declaration of Helsinki and must have been approved by an appropriate ethics committee. A statement detailing this, including the name of the ethics committee and the reference number where appropriate, must appear in all manuscripts reporting such research. If a study has been granted an exemption from requiring ethics approval, this should also be detailed in the manuscript (including the name of the ethics committee that granted the exemption). Further information and documentation to support this should be made available to Editors on request. Manuscripts may be rejected if the Editor considers that the research has not been carried out within an appropriate ethical framework. In rare cases, Editors may contact the ethics committee for further information.

Experimental research on vertebrates or any regulated invertebrates must comply with institutional, national, or international guidelines, and where available should have been approved by an appropriate ethics committee. A statement detailing compliance with guidelines and/or ethical approval must be included in the manuscript. For studies involving client-owned animals, authors must document informed client consent and adherence to a high standard (best practice) of veterinary care.

This article ☐ **does** ☑ **does not**
contain any studies with human or animal subjects performed by any of the authors.

7. Informed Consent.

All individuals have individual rights that are not to be infringed. Individual participants in studies have, for example, the right to decide what happens to the (identifiable) personal data gathered, to what they have said during a study or an interview, as well as to any photograph that was taken.

Hence it is important that all participants gave their informed consent in writing prior to inclusion in the study. Identifying details (names, dates of birth, identity numbers and other information) of the participants that were studied should not be published in written descriptions, photographs, and genetic profiles unless the information is essential for scientific purposes and the participant (or parent or guardian if the participant is incapable) gave written informed consent for publication. Complete anonymity is difficult to achieve in some cases, and informed consent should be obtained if there is any doubt.

If identifying characteristics are altered to protect anonymity, such as in genetic profiles, authors should provide assurance that alterations do not distort scientific meaning.

The following statement should be included:

**Topic**	**Statement**
Informed consent	Informed consent: Informed consent was obtained from all individual participants included in the study.
If identifying information about participants is available in the article	Informed consent: Additional informed consent was obtained from all individual participants for whom identifying information is included in this article.

I hereby certify that the information set forth above is true and complete to the best of my knowledge.

Signature: Christianson, Amy Digitally signed by Christianson, Amy Date: 2021.06.17 15:28:02 -06'00'

Date: June 17, 2021

Definitions.

Other: Anything not covered under the previous three boxes

Pending: The patent has been filed but not issued

Issued: The patent has been issued by the agency

Licensed: The patent has been licensed to an entity, whether earning royalties or not

Royalties: Funds are coming in to you or your institution due to your patent

Entity: government agency, foundation, commercial sponsor, academic institution, etc.

Grant: A grant from an entity, generally [but not always] paid to your organization

Personal Fees: Monies paid to you for services rendered, generally honoraria, royalties, or fees for consulting, lectures, speakers bureaus, expert testimony, employment, or other affiliations

Non-Financial Support: Examples include travel paid by the entity, writing assistance, administrative support, etc.

At the time of manuscript acceptance, journals will ask authors to confirm and, if necessary, update their disclosure statements. On occasion, journals may ask authors to disclose further information about reported relationships.
